# Exploring the Spatio-Temporal Dynamics of Reservoir Hosts, Vectors, and Human Hosts of West Nile Virus: A Review of the Recent Literature

**DOI:** 10.3390/ijerph10115399

**Published:** 2013-10-25

**Authors:** Esra Ozdenerol, Gregory N. Taff, Cem Akkus

**Affiliations:** 1 Department of Earth Sciences, University of Memphis, Memphis, TN 38152, USA; E-Mail: cakkus@memphis.edu; 2 Norwegian Forest and Landscape Institute, Tromso 9007, Norway; E-Mail: gta@skogoglandskap.no

**Keywords:** West Nile Virus, geographic distribution, risk modeling

## Abstract

Over the last two decades West Nile Virus (WNV) has been responsible for significant disease outbreaks in humans and animals in many parts of the World. Its extremely rapid global diffusion argues for a better understanding of its geographic extent. The purpose of this inquiry was to explore spatio-temporal patterns of WNV using geospatial technologies to study populations of the reservoir hosts, vectors, and human hosts, in addition to the spatio-temporal interactions among these populations. Review of the recent literature on spatial WNV disease risk modeling led to the conclusion that numerous environmental factors might be critical for its dissemination. New Geographic Information Systems (GIS)-based studies are monitoring occurrence at the macro-level, and helping pinpoint areas of occurrence at the micro-level, where geographically-targeted, species-specific control measures are sometimes taken and more sophisticated methods of surveillance have been used.

## 1. Introduction

The resurgence of interest in West Nile Virus (WNV) no doubt reflects its emergence and reemergence in populations and areas of the World where infections have previously been rare. The study of new strains has become a considerable focus of research activity, especially, when the virus gained publicity in 1999 in the U.S., after its first appearance in the Western Hemisphere [1]. The purpose of this inquiry was to explore new strains and their incursion into new geographic regions. This review discusses the epidemiology of WNV, its dissemination across the world, and summarizes ecological studies conducted over the past decade that have used a variety of sophisticated methods of surveillance and spatial disease risk modeling using Geographic Information Systems (GIS) and remote sensing (RS).

### 1.1. WNV Epidemical History and Geography

WNV is a mosquito-borne zoonotic arbovirus belonging to the genus Flavivirus in the family Flaviviridae. Flavivirus is closely related to Japanese encephalitis virus in Eastern Asia; Kunjin virus, in Australia and Southeast Asia and St. Louis encephalitis virus, in North and South America [1,2]. Birds are the main reservoir hosts of WNV, and mosquitoes are the main vector for the virus transmission from birds to humans, horses, and other birds. WNV epidemics mainly occur in summer and autumn in temperate, subtropical, and tropical areas. Because WNV is highly influenced by regular, seasonal climate, and environmental changes, it is particularly amenable to spatial and temporal analysis [2]. During the mosquito season, mosquitoes become infected with the West Nile Virus primarily through bird-blood meals and then retransmit the virus to any one of multiple bird species, a cycle which amplifies the virus. Governed by environmental conditions and host behaviors, infected mosquitoes can spread WNV to other incidental hosts, such as humans and horses. Endemic to Africa, Asia, Europe, Australia, and now the Caribbean and the Americas, the origin of the original strain of WNV is the Middle East, but the mode of introduction is unknown [3]. Since its discovery in Uganda in 1937 [4], the geographic distribution of West Nile Virus has expanded. Outbreaks occurred in Israel in the 1950s, in southern France and Russia in the early 1960s, and in South Africa, Belarus, and Ukraine in the 1970s [5,6]. After two silent decades, several human and equine outbreaks of fatal encephalitis occurred from 1996 to 2000 in Romania, Czechoslovakia, Morocco, Algiers, Tunisia, Italy, Russia, Israel, and France [7,8,9]. Other subsequent outbreaks have been observed in Azerbaijan, Central African Republic, Democratic Republic of Congo, Egypt, Ethiopia, India, Madagascar, Nigeria, Pakistan, Senegal, Sudan and several European Countries [10]. Serologic evidence (diagnostic identification of antibodies in serum) exposure to WNV has also been detected in humans in Armenia, Borneo, China, Georgia, Iraq, Iran, Kenya, Lebanon, Malaysia, the Philippines, Sri Lanka, Sudan, Syria, Thailand, Tunisia, and Turkey [11,12,13,14,15].

In India, the first WNV encephalitis human case was reported from Bombay in 1952 [16]. Recently, 88 serologically confirmed human cases were reported in 2002. In India, fatal cases were seen in children, unlike the almost exclusively older age groups seen in other countries [17]. Japan’s first WNV case was confirmed in September, 2005 which involved a patient who had returned to Japan from the United States [18]. Recently, WNV meningitis/encephalitis was observed in Xinjiang, China in 2004 [19]. Since then, China has taken public health measures to prevent the virus from being introduced there. No evidence of WNV activity was found after a nationwide surveillance of dead wild birds during 2005–2008 in South Korea [20]. In October 2012, a returning traveler from Africa was the country’s first West Nile virus case [21].

According to phylogenetic studies (study of evolutionary relationships among species), there are two main lineages of WNV strains. Strains from lineage I cause the outbreaks in Europe and in the Mediterranean basin and is endemic in Africa, India and Australia. Strains from lineage II are only present in sub-Saharan Africa. In 1998, a surveillance study of dying migrating storks and domestic geese identified a virulent WNV strain from lineage I in Israel [22]. During the prolonged spring drought and heat wave of 1999, a nearly identical WNV strain suddenly emerged in New York, killing native birds and causing fatal cases in humans. In 2002, the virus was spread to 44 states, Washington D.C. and five Canadian provinces. Claiming 284 cases, the largest outbreak in the Western Hemisphere was recorded that year [23]. Intense research efforts took place to identify the particular strains involved in the United States [24,25,26].

Over the five years following 2002, the virus spread across the continental United States, north into Canada, and southward into the Caribbean Islands and South America. Over the last decade, in Europe, the disease incidence has increased in areas where it had already been reported and, most recently, affected other areas where it had never been observed before (Ireland, Sardinia, Marche, and Greece) [27,28]. In 2004, first reported cases of imported WNV infection in northwest Europe was acquired by Irish tourists travelling in Portugal [29]. The rediscovery of *Culex modestus*, a species of mosquito capable of carrying WNV (first discovered in 1945) in 2012 in Kent and Essex regions of the United Kingdom drew considerable attention to the virus because of the potential risk of its spread [30]. Furthermore, In Ireland and the United Kingdom, information has been produced for travelers to Southern European Countries where sporadic WNV activity has been reported over the last 40 years [31,32,33].

In the United States, WNV spread from east to west, but since 2001, the Southern states have usually been affected earlier in the season than the North because of the warmer weather [34]. The recent unusually mild winters, early springs, and early summers have aided transmission of the virus in Texas in the summer of 2012. After seeing the worst toll from West Nile, Texas declared a state of emergency, which has reached 270 cases and 11 deaths in Dallas County alone [35]. The increasing occurrences of warmer and wetter weather patterns in the northern United States have increased the mosquito life span leading to increased incidence of WNV infections [36].

To limit the impact of the virus in the United States, the CDC started active bird surveillance in wild and sentinel populations, active mosquito surveillance to monitor virus activity and to identify potential vectors, and active veterinary surveillance particularly for horses and passive human surveillance for reporting viral encephalitis [37,38]. Vector and host surveillance and analysis of the ecology of infectious diseases in animal populations became essential components of public health programs to address WNV epidemics. Animals play many roles in transmitting disease to humans. WNV has spread to 230 species of animals, including 138 species of birds. Since the New York outbreak had resulted in extensive mortality in crows, this led to increased research on the links between bird migration patterns and the spread of the WNV epidemic [1].

The southward dissemination of WNV into the Caribbean and Central and South America is attributed to migratory birds [39]. The virus was first detected in 2001 in Jamaica and the Cayman islands. Cross-reactive WNV antibodies in humans have been detected in Mexico, the Bahamas, and Cuba [40]. Serologic evidence of WNV infection in horses was reported in Guadalupe, Mexico, Central America, Puerto Rico, and Colombia [40]. Resident birds were tested positive for antibodies to WNV in the Dominican Republic and Venezuela [40]. In 2006, four serologically confirmed human WNV encephalitis cases were reported in Argentina [41]. In Brazil, antibodies to WNV were reported in Santa Caterina state bordering Argentina, but no human infections have been reported [42,43]. The explanation for the paucity in WNV detection in tropical America may be the lack of WNV surveillance. Presently, WNV public health impact remains poorly understood in the Caribbean basin and South America [40].

**Figure 1:**
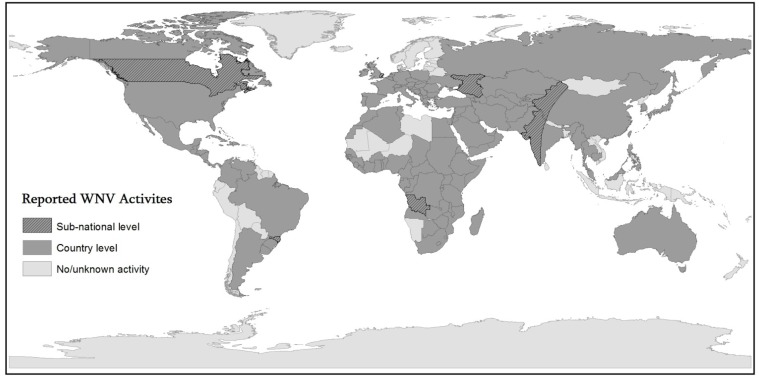
Reported WNV activities.

Figure 1 presents the geographic dimension of the current distribution of WNV activity in the world. The dark hatch pattern represents areas in which WNV activity has been reported at the sub-national level in particular regions of some countries and the lighter gray shading signifies countries with (at least) some reported WNV activity, and the lightest gray represents counties with no or unknown activity. Our review shows a wider spread in comparison to the previous review published in 2002 [44]. 

### 1.2. Ecological Studies

Ecological studies of agent-vector-host relationships and improved surveillance methods have been cited as important priorities for addressing WNV epidemics, monitoring WNV spread, and predicting future outbreaks [45,46,47,48,49,50,51,52,53]. Ecological studies are also valuable because they may give insight into factors that affect temporal cycles in infectious disease risk [54,55]. An increasing number of GIS-based ecological studies have used data of host animals to investigate vector distribution. For example, studies have attempted to model the distribution of infected pools of birds, horses, and mosquitoes. These studies are obviously limited by spatial biases where hosts are killed or died [45,46,47,48,56]. Local responses have utilized GIS technology, particularly in bird surveillance. The geocoded locations of dead wildlife were plotted with test results of WNV and evidence that the virus remained in bird populations over the winter reinforced the importance of preparations for the following spring and summer [57].

To predict and explain the spread of WNV, one needs to understand the complex interrelationships among human, avian, and mosquito habitat systems and the inter-play of environmental, built-environment, and anthropogenic risk factors that influence these systems [45,46,47]. Both prospective and retrospective techniques are needed to identify WNV exposure areas [58]. Prospective techniques are mainly early warning approaches based on information on a single component of the WNV transmission cycle, *i.e*., either infected birds or human cases. Retrospective techniques are spatial statistical modeling approaches, relating the incidence cases of either infected dead birds or human cases to a range of risk factors. It is vital to incorporate the spatial-temporal information of the three components of the transmission cycle affecting virus spread—birds (reservoir), mosquitos (vector), and humans (host)—in the model, to understand the viral transmission. Habitat studies of environmental risk exposure needs to be validated by models using follow up data on the distribution of human cases and vectors otherwise modeling environmental risk based on habitat alone could be problematic. The geographical distribution of risk areas (based on environmental conditions) needs to be compared to the distribution of vectors and reservoir hosts and human cases [59].

GIS and Remote Sensing (RS) are useful techniques to model habitat since the likelihood of producing a map of infected vectors or hosts for all locales could be difficult. Remote sensing models have been used to define suitable landscape features and meteorological variables for vector transmission and potential vector and host mechanisms of dispersal in many parts of the world [59,60,61,62,63]. Environmental data from Remote Sensing such as vegetation type and density, elevation, slope, hydrology, and soil moisture have been used to model host risk exposure. Consequent GIS analysis could quantify associations between risk variables and environmentally-sampled covariates [60,61,62,63,64,65]. Time series overlay analyses of thematic geographic data and spatial intersection analyses; buffer generation and neighborhood analysis; vector-borne grid generation and network analysis; and disease risk surface modeling are other common data analysis and functions using GIS/RS technology [66,67]. Geographic Information Systems (GIS) are being used for proactive surveillance, prevention, and control measures and to study patterns of environmental factors such as global climate change and their possible impacts on the spread of WNV [61,68,69,70]. GIS and RS are also employed by Centers for Disease Control (CDC) and United States Geological Survey (USGS) to prepare interpretive maps showing WNV activity in North America [71]. 

Increased international travel and exchange of goods—including animals—have an impact on geographic patterns of WNV. Global trade and modern transportation technology have made it possible for translocation of species and for individuals to contract a disease in one place and be in another country by the time symptoms appear. Numerous studies explored travel patterns and their association with dissemination of the virus [72,73,74,75,76,77]. These studies concluded that the risk of infection to travelers cannot be quantified at present, but the risk is related to exposure to mosquito bites. It is important that travelers to areas where there is active transmission to humans of West Nile virus by mosquitoes, particularly persons who are over 50 years old or those with immunosuppression are aware of the potential risk and adopt mosquito-bite prevention measures. The ability to diagnose WVN infection requires health care professionals to consider travel locations of returning travelers from endemic areas presenting with encephalitis or meningitis [77].

### 1.3. Recent Reviews

Twenty-five review articles were published since the New York outbreak in 1999, when WNV was at the forefront of public health concerns [11,12,13,14,15,78,79,80,81,82,83,84,85,86,87,88,89,90,91,92,93,94,95,96,97,98]. This explosion of reviews has focused mostly on topics from virology and genetics to non-vector transmission, clinical features, diagnosis, prevention, or vaccination. Bledsoe, Garmendia, Campbell, and Granwehr [11,96,97,98] reported a few studies with GIS techniques and surveillance methods. Clements *et al*. [98] had a more extensive review of spatial methods and applications of spatially-explicit models to ecologic studies of emerging viral diseases but not inclusive of WNV. Our updated review focuses on spatial studies applied specifically to WNV.

In his review in 2001, Garmendia *et al*. [96] gave a historical overview of the spread of the virus until its emergence in the northeastern United States and summarized studies which confirmed the establishment of WNV endemically in the United States. The authors summarized surveillance results and spread of the virus to several northeastern states. They showed the distribution of West Nile fever in the years 1999 and 2000 in northern states by mapping related human, bird and horse deaths. Germandia *et al*. further explained the CDC’s measures to prevent the infection and limit its spread in the United States. A national West Nile Virus Surveillance System in year 2000 was planned and executed by a coordinated effort of the Department of Human Health and Services, the USGS, the CDC, the United States Department of Agriculture (USDA), state public health departments, and natural resource agencies. The CDC released surveillance funds for mosquito testing and the testing of sentinel chickens, wild birds, domestic animals, and humans with neurologic disease for WNV. The findings from the Germandia *et al*. review provided a new basis for future evaluations [96].

The second review by Campbell *et al*. [44] in 2002 examines and illustrates the extended range of the WNV in the western hemisphere. They determined that the prevalence of immunity to WNV depends on geography and the human population studied. They explained the epidemic vectors of the virus by region and they explained that the mosquito-bird-mosquito transmission cycle involved primarily the *Culex* mosquitoes. They concluded their review with a summary of the poorly understood ecological and climatological factors of the epidemic and provided a new basis for future studies.

The review by Granwehr *et al*. [97] in 2004 provided an update on knowledge of WNV biology that can be used to highlight the advances in the field and development of control measures. The westward expansion of the epidemic at the US level has been mapped based on non-human WNV activity and human case occurrences. A key issue in predicting the spread of the virus, and a major issue brought up in the review, is whether WNV will become endemic or epidemic, and they concluded either outcome is possible.

Bledsoe’s [11] review in 2004 is the first one reporting the importance of spatial decision-support systems (SDSS) [99] which integrate GIS and epidemiologic databases to facilitate visualization, querying and interpretation of spatial epidemiologic datasets [99,100]. He summarizes inventive strategies for mosquito control and lists larvicides and adulticides used for mosquito control approved by the US Environmental Protection Agency (EPA). His review particularly contributes to prevention and public health measures and lessons learned regarding the virus.

Clements *et al*. in 2009 [98] categorized studies under four main topics: landscape epidemiology, phylogeography, cluster detection, and early warning systems and spatially-explicit statistical models. He defines landscape epidemiology as a sub-discipline integrating epidemiological data with landscape data (climatic, topographical, remote-sensing-derived land cover data including standard measures of vegetation cover such as Normalized Difference Vegetation Index—NDVI). Under landscape epidemiology, they reported studies that used ecological niche models, which are a class of statistical algorithms to predict, in this case, WNV vector and reservoir habitats based on landscape data, and these studies then map their potential distributions [101,102,103,104,105,106,107]. These studies reported associations between vector abundance and various landscape elements such as temperature, precipitation, NDVI, urban landscape features, wetland, and vegetation cover. Clements *et al*. [98], in their review, also touched on fuzzy methods such as Multiple Criteria Decision Analysis (MCDA), combining the environmental suitability probabilities of a location for disease presence across multiple determinants such as vegetation cover, slope, elevation, and distance to aquatic habitats and a weighted linear combination (WLC), a weighted suitability method across multiple determinants. In their review, they promoted these methods as highly applicable for decision making in resource-allocation and risk management [98,108].

Use of other widely applied models (regression models) and statistical analysis (principal components analysis and discriminant analysis) in landscape epidemiology were reported [47,109,110]. In these models, RS- and GIS-derived environmental variables measured at sampled locations are entered as covariates and the resultant model is used to predict the outcome variable (vector abundance, presence of disease). For example, associations with drought and land surface wetness prior to time of transmission were found in Florida and subsequently WNV epidemics were forecasted according to variation in water table depth [109].

Clements *et al*. [98] reported a phylogeographical study of WNV (spatial analysis of genetic variation) by Betoletti *et al*. [111], which found little geographical but significant temporal variation in the U.S., with increasing viral diversification from 2002 to 2005. With their review, they suggested future applications to integrate phylogeographic analysis with spatial statistical modeling to better quantify and predict disease distributions.

Clements *et al*. [98] also reported studies that utilized spatial [102,103] and spatiotemporal clustering techniques [51,112] to detect and characterize clusters of cases of WNV. These techniques include *K*-function [113], the Cuzick and Edwards test [114], Knox’s test [115], and the spatial scan statistics [116]. They concluded that these techniques are proficient in detecting spatiotemporal clusters in real-time and provide timely interventions in delineating high-risk areas.

Clements *et al*. [98] also reported on the West Nile Virus Information System (WeNiVIS) [117] and the integrated system for public health monitoring of West Nile virus (ISPHM-WNV) [118] in their review. These internet-based Spatial Decision Support Systems (SDSS) provide visual analysis of the distribution of WNV in North America, and include spatial and temporal querying capabilities.

The rapid global diffusion of WNV argues for a better understanding of its geographic extent. The number of ecological studies that have used GIS and spatial modeling has increased significantly over the past decade, examining climate factors in addition to biological and ecological determinants, which are strain-dependent. Moreover, with the availability of vector geographic data and local response involving GIS and remote sensing technologies, particularly in bird and mosquito surveillance, comparability across studies is enhanced. The purpose of this study is to review the state of spatio-temporal interactions of reservoir hosts, vectors, and humans on multiple time and spatial scales. As outbreaks of WNV remain unpredictable, further coordinated studies are needed for a better understanding of the ecology and the geographic distribution of the West Nile Virus. Finally, in light of new evidence, suggestions for future studies are proposed.

## 2. Methods

A literature research was conducted to identify recent articles that examined new strains and their incursion into new geographic regions. Spatial modeling of WNV infection and risk factors that affect temporal cycles were of particular concern. Several online databases were queried, including PubMed, ScienceDirect, Web of Science, and Questia. The following terms were used individually and in combination to source articles for consideration: West Nile Virus, new strains of West Nile Virus, vector surveillance, mosquito vectors, avian GIS models, monitoring of West Nile Virus, West Nile Virus outbreak, geographic distribution of West Nile Virus, GIS and RS applications and West Nile Virus. Our review covers a nearly 14 year period, inclusive of GIS-based studies published since a new strain was first identified in New York City in 1999. Initial searches yielded approximately 51 results. The abstracts of these papers were reviewed to confirm applicability. After considering additional exclusion criteria (non-English language, manuscripts not available as full-text), 47 papers remained. Since we reviewed studies related to exposure areas, spatial modeling and surveillance methods, and transmission cycles retrospectively in their entirety (*i.e*., birds, mosquitoes and humans), articles were summarized and grouped into nine categories: spatial analysis of human cases, birds, mosquito pools, equine cases (four), habitat-based studies, RS-based early warning systems and real-time GIS studies (two) and spatial analysis of genetic variation and uncertainty (two). Table 1 presents these studies under each category with GIS methods applied, study region and date and resultant common risk factors with location-dependent ones indicated (Table 1).

## 3. Results and Discussion

### 3.1. Spatial Analysis of Human Case Incidence

Ten articles examined spatial clustering of human case incidence [45,55,58,64,119,120,121,122,123,124]. A study of the 2002 outbreak in Chicago region by Ruiz *et al*. [45] defined the conditions that distinguish places with high focused incidence of human cases from places where few or no human cases were reported. Local Moran’s I, discriminant analysis (DA), and binary logistic regression models were developed to find disease clusters and determine environmental and social predictors of WNV cases.

**Table 1:** Summary of studies with common risk factors.

Analysis/Citation	Region/Date	Common Risk Factors (* Location-Dependent)
*Spatial Analysis of human case incidence*		
Local Moran’s I [45]	Chicago, 2002	Less Population density *, higher percent of old and white residents *, poor drainage, mosquito abatement efforts
SaTScan, Local Moran’s I [55]	U.S. level, 2002–2008	Study focused on hot-spots of human case incidence
Conditional Autoregressive Model [58]	U.S. level, 2013	The number of WNV positive mosquito pools
Global Moran’s I [64]	U.S. level, 1999–2008	Temperature and precipitation ranges
Ripley’s K test [119]	Chicago, 2005–2006	Inner suburbs, less densely populated areas *, high percent of white residents *, post world war II housing and a higher median population age, smaller elevation ranges, standing water, more vegetated areas
Hot spot analysis [120]	Connecticut, 2000–2005	Urban/suburban areas
Spatial proximity, Moran’s I [121]	Northeast U.S.	Urban/suburban areas, less forested landscapes
Global Moran’s I, NDVI [122]	Iowa, 2003–2006	Less population density * and rural agricultural areas, drier conditions
SaTScan [123]	Northern plains, 2003	Rural areas, irrigated land in rural areas
SaTScan, Local Moran’s I [124]	Davis, CA, USA, 2006	Avian mortality, residential landscape, warm night temperatures
Moran’s I [125]		Spatial autocorrelation and contagious diffusion
*Spatial-temporal analysis of bird species*		
NND Time Model [36]	Twin Cities, 2002	Densely populated areas * , distance to nearest dead bird and pool location
Mapping migration routes [57]	North America	Wintering grounds along coastal plains of Georgia, northern Florida
Kriging [62]	Indiana, 2002	High temperatures in August-September months
Bird abundance mapping [69]	British Columbia, 1994–2003	Dead corvid density
Proximity analysis [102]	Texas, 2002	Proximity of equine cases to human cases in urban populations
GLMM [126]	Alberta, Canada 2002–2006	The grassland natural region, rural/suburban areas
Discriminant Analysis, Mahallanobis DS [127]	Virginia, 2011	Mean precipitation, percent impervious surface with 21–40% canopy density
Mahallanobis Distance Statistics [128]		Shorter distance to bird risk areas associates with higher risk
*Spatial analysis of horses*		
Kriging [61]	Indiana, 2002	High temperatures in August-September months
Sptiotemporal clustering, NDVI analysis[102]	N. Indiana, 2002	High median estimated NDVI in equine clusters
Proximity analysis[129]	Texas, 2002	Proximity of equine cases to human cases in urban populations
LULC analysis, SatScan clustering [130]	France	Rice fields, dry bushes, open water, low elevation salted swamps
SaTScan [131]		Hot spot analysis, Cluster identification
SaTScan [132]	Texas	Study focused on areas-of-high-risk
*Spatial modeling of mosquito pools*		
Risk mapping [46]	Mississippi	High road density, low stream density and gentle slopes
Mahallanobis Distance Statistics [53]	Tennessee, 2004	High percentage of black population, low income, high rental occupation, old structures, vacant housing
Spatial sensitivity analysis [63]	Colorado, 2003–2007	Study focused on sub-county scale presentation and how WNV disease occurence influenced by data aggregation
Spatio-temporal spread, risk mapping [133]	Australia, 2013	Predictive risk-zone mapping
*Real-time GIS Studies for WNV surveliance*		
ArboNet, CDC [37]	U.S.	Real-time GIS study for WNV. surveiliance, prevention and control
WNV-Multi Agent Geo-Simulation [70]	Quebec, Canada	Short-term decision making related to use of larvicides with climatic scenarios
ISPHM-WNV [118]	Quebec, Canada, 2002	Real-time GIS study for public health surveiliance
Real-time GIS-driven Surveilliance [134]	Canada	Real-time GIS driven surveilliance pilot system
A nationwide electronic surveilliance [135]	Canada	A nationwide electronic surveilliance
*Habitat-based Studies*		
LULC analysis [2]	Saskatchewan, Canada, 2003–2007	Study focused on risk mapping
Maximum likelihood unsupervised classification LULC change matrix [39]	Urbana Champaign, IL, USA, 1991–2003	Residential high canopy coverage
Generation of DEM, Spatial Hydological Modeling, Eigen vector mapping [40]	Trinidad, 2008–2009	Terrain elevation
Raster-based mosquito abundance model [48]	British Columbia	Study focused on risk prone areas
Geospatial models based on LULC [60]	Cook County, IL, USA, 2002–2005	Warmer temperature and heavy precipitation, forest and middle-range built environment
Terrain Analysis, ISODATA [61]	Tuskegee, AL, USA	Smaller elevation range
Shortest distance analysis [136]	17 U.S. States, 2001–2005	Warmer temperatures, elevated humidity and heavy percipitation
NDVI analysis, RS-driven spatial analysis [137]	Morocco	Precipitation
Computational neuronetworks [138,139]	Twin Cities, MN, USA, 2002–2006	Proximity to wetlands
*RS Studies for early warning systems*		
ASTER imagery and high-temporal MODIS [127]	N. Virginia	Elevation and urban built-up conditions negatively correlated with WNV propagation, landsurface temperature positively correlated with viral transmission
ASTER imagery and high-temporal MODIS [140]	Indiananpolis	
ASTER imagery and high-temporal MODIS [141]	Chicago	
AMSR-E dervied models [142]	South Dakota	Air temperature and vegetation opacity and surface water fraction
Tassled-Cap transformation [143]	Coastal Virginia	Study focused on developing a habitat suitability index
AVIRIS [144]	Fresno, Canada	Neglected swimming pools
NDWI [145]	Atlanta, GA, USA	Neglected swimming pools
*Spatial analysis of genetic variation*		
Population genetic analysis [111,146,147]	U.S. level	Localized environmental conditions
Population genetic analysis [148]	Chicago, 2008	Seasonal variations in microclimatic conditions at finer scale
Spatial uncertainty analysis		
Spatial uncertainty analysis, SaTScan [149]	South Dakota	Lower ability to geocode Indian reservations

In the Chicago region the *Culex pipiens* mosquito is of special concern as a key WNV vector species. The results revealed that a tract is more likely to include at least one case when it has lower population density, is relatively close to bird specimens, is comprised of a higher percentage of older and white residents, and has a higher percentage of housing built between 1950 and 1959. The Chicago Lake Plain region was a high risk area with poor drainage and housing built in the post-World War II era with catch basins rich with organic material, and therefore proved an excellent breeding ground for *Culex* mosquitos. The high income of that area was a factor in more cases being reported, while at the same time may have resulted in more outspoken protest to mosquito control efforts (spraying), making more obstacles for efficient and early control.

Sugumaran *et al*. [55] combined two spatial statistics, Kulldorff’s spatial scan statistic to find space-time clusters of WNV cases (using SaTScan software) and Anselin’s Local Moran’s I statistic (using ArcGIS), to reveal significant spatial clusters of human WNV incidence in the continental United States. They determined that between the years of 2002 and 2008, there was significant clustering of human WNV incidence each year. The results of these statistics and clusters illustrate where important WNV hot-spots are located throughout the country.

Messina *et al*. [119] drew on the study by Ruiz *et al*. [45] and investigated the patterns and risk factors of WNV in the Chicago area in subsequent years to determine if the same patterns found in 2002 (nonrandom pattern) were similar to subsequent years, including the next largest outbreak years of 2005 and 2006. They included precipitation and mosquito infection data which were not available for the earlier analysis. The Ripley’s K test showed global spatial clustering of individual case locations in 2002, 2005, and 2006 across all distances up to 30 km. Results for the global Moran’s I statistic showed global spatial clustering of age-adjusted incidence rates for census tracts in some but not all study years. Based on the risk maps from the regression models, the Chicago city center showed the lowest risk across the years, with the inner suburbs showing the greatest risk for 2002 and the western most part of the study area showing greatest risk in 2005 and 2006. Comparing all six regression models revealed that while risk for human WNV infection has persisted in predominantly white and less densely populated areas since 2002, a different combination of factors was found to be significant in each of the subsequent outbreak years. Areas with more post world war II housing and a higher median population age experienced greater risk in the first two outbreak years, and drier, less diverse areas experienced greater risk in later years. Neighborhoods with smaller elevation ranges were at increased risk in the largest outbreak years of 2002 and 2005 indicating census tracts relatively flatter than others may have more places for the accumulation of standing water needed for *Culex* breeding. More vegetated areas saw greater risk in the 2006 outbreak. The review concluded that more attention should be given to the significant positive relationship between median income and WNV risk in 2005 and 2006. This study provided an example of how disease event data and publicly available census and environmental variables could be combined with spatial analytical methods to lead to new information about the spread of a vector-borne disease.

Brownstein *et al*. [58] used a conditional autoregressive model to create an early warning system for human risk to the virus. They calculated expected WNV incidence rates using USGS West Nile maps and Census 2000 population data and were able to create an expected human incidence map across the continental United States early in the transmission season. They validated their model-estimated risk map *versus* the raw incidence map from 13 August for predicting the case distribution for 1 October 2003. Based on the actual cases recorded on October 1, their model predicted a fairly accurate incidence of cases from 13 August. Their results suggested that this model could be used to prevent human cases before they emerge. They also determined that there is a stronger positive relationship between human WNV cases and the number of WNV-positive mosquito pools than for human cases and WNV-positive dead birds.

Liu *et al*. [120] performed a multi-year analysis of risk factors for WNV infection in humans in the state of Connecticut for the years 2000–2005 to determine the best predictive model for human WNV infection over this time period and explored whether the patterns of risk factors were changing. They mapped the hotspots of human infection risk by a set of static variables (land use and population density) and dynamic environmental variables such as statewide climate data (daily temperature, yearly precipitation, growing degree days) and animal sentinel data (dead bird sightings, WNV positive birds, WV positive mosquitoes, Mosquito abundance). They concluded that a real-time model using climate, land use, and animal surveillance variables to predict WNV risk appeared feasible. In fact, their study considered all variables and concluded that population density, growing degree days, temperature, WNV positive mosquitoes, dead birds, and WNV positive birds were significant factors determining human infection risk. They found positive associations between human infection and urban/suburban environments *versus* more rural areas [47,121]. While their findings agreed with other studies [47,121] they also contradicted with studies such as Degroote *et al*. [122] and Wimberly *et al*. [123] who suggest the opposite is true in that less population density and rural areas is a risk factor for the West Nile disease. Liu *et al*. attributed this discrepancy to the geographical region where these studies were conducted with some being in the Eastern United States [47,120] and the others in the Western United States [122,123]. The importance of climatic factors in Liu’s model were in agreement with reports by Gosselin *et. al* [118] and Nielsen [124] suggesting real-time climate data can be useful in WNV risk prediction.

Young and Jensen [64] recently conducted a nationwide study at the county level from 1999–2008 examining spatial autocorrelation and clustering of WNV human cases. They obtained cases from CDC’s ArboNet system, a system where state health departments upload confirmed cases as non-neuroinvasive and neuroinvasive. They did their analysis by using both normalized and non-normalized data. The normalized data were normalized by county population and multiplied by 100,000. They used the Moran’s I measure to compute spatial autocorrelation statistics [125]. They plotted Moran’s I z-scores for both neuroinvasive, non-neuroinvasive, and total human cases, and found positive spatial autocorrelation in every single year, with exceptionally high scores the first years WNV was introduced in New York State. They also found WNV was slowly spreading to conterminous United States through contagious diffusion, and that WNV infection is related to temperature and precipitation ranges. Regions with larger precipitation variations had stronger Moran’s I values.

### 3.2. Spatial-Temporal Analysis of Bird Species

Five studies addressed high-risk areas for WNV exposure to humans by focusing primarily on the measured presence of WNV-positive bird specimens. Rappole *et al*. [57] presented an overview of the association of WNV with birds, focusing in particular on the advent and movement of the virus in the Western Hemisphere. They compared the translocation of the virus by migratory birds in the Old World and in the New World settings. In the New World, zoo, pet, domestic and wild birds were responsible for virus introduction. The human outbreaks happened at urban locales such as zoos and near wetlands. The Old World epidemics of WNV had few concurrent reports of deaths of infected birds. Rappole *et al*. defined three possible modes of entry for transatlantic migration of avian species; pet and domestic bird trade, migration and vagrant. They also presented a comprehensive list of avian species of transatlantic migration from Euroasia to the eastern United States. They predicted that storm-transported birds would be infected with the West African form of the virus rather than the Middle Eastern one. They also mapped migration routes of species that breed in temperate North America by: Circum-Gulf Route, Trans-Gulf Route, and the Caribbean Island/Western North Atlantic Route. They mapped the migration patterns by band returns, banding locations, and recovery locations. Banding data, showing winter concentration areas for large numbers of birds indicated that the most likely place for bird-spawned outbreak on the wintering ground would be along the coastal plain of Georgia, northern Florida, or Alabama.

David *et al*. [69] conducted a spatial study to determine whether the system was accurately portraying corvid mortality rather than displaying regional differences in surveillance methods. To find out whether reports of dead corvid sightings and submissions of dead corvids for WNV were representative of true corvid mortality in British Columbia, they used Breeding Bird Survey (BBS) bird abundance map data for the period of 1994–2003 to estimate corvid density in each local health area (LHA). Some local areas were overrepresented (78% met or exceeded expectations for reports of dead corvid sightings) and other under-represented (5%) in terms of corvid WNV surveillance indicators. Recommendations were made to improve the representativeness of corvid surveillance data. They concluded that rural areas with low corvid densities may need to focus on other methods of WNV detection. Dead corvids for WNV testing were representative of true corvid mortality in British Colombia.

Yiannakoulias *et al*. [126] analyzed the relationship of several factors, including infected dead birds, to human WNV incidence in 503 Alberta cities, towns and villages in Canada that have a population greater than 100 people. Infected bird data were acquired through a passive surveillance system administered by Alberta Sustainable Resource Development in which the public turned in dead birds. They incorporated irrigation, ecological and urban/rural information into a regression model. They used a variogram to visualize the magnitude of spatial dependence in the residuals of the non-spatial model. To manage the effects of residual spatial autocorrelation, they used generalized linear mixed models (GLMM) to model the incidence of infection. Infected bird data contributed little to their model. They concluded that WNV was more common in Southeastern Alberta in 2003. The grassland natural region supported *Cx. tarsalis*, a foul-water preferring species found in rural and suburban, rather than urban areas.

Using data from the Twin Cities Metropolitan Area of Minnesota (TCMA) for the years 2002 to 2006, Ghosh *et al*. [36] introduced the Nearest Neighbor Distance Time (NNDT) method for delineating WNV exposure areas and WNV transmission cycles. The NNDT method retrospectively identified WNV transmission cycles with an area ranging from 5 to 150 square miles. NNDT calculated distance to the nearest dead bird or pool location, created spider diagrams for these nearest neighbors, and selected spider diagrams centered on zip codes that satisfy the spatial and temporal rules to delineate WNV transmission cycle. After 2002, the location of the cycles shifted from the suburban western and southwestern TCMA to the densely populated areas near the core cities of Minneapolis and Saint Paul. The cycles peaked 10–15 days prior to the human outbreaks. NNDT demonstrated how examining distances among the locations of infected dead birds, positive mosquito pools, and infected human cases infuses a local dimension to the global knowledge of the time required for the virus to be transmitted from bird to mosquito and then to humans in the cycle. In TCMA, the cycle peaked 10–15 days prior to the onset of human illness, a period which was consistent with the epidemiology of the virus transmission from bird to mosquito and then to humans.

A recent study by Liu *et al*. [127] used a battery of techniques (discriminant analysis, Mahalanobis distance statistic) to assess the influence of levels of urbanization and canopy density on bird infection and WNV transmission in northern Virginia. With the use of RS and GIS, they classified percent impervious surface into five classes ranging 0–20%, 21–40%, 41–60%, 61–80%, and 81–100%, and four classes for percent canopy density, 0–20%, 21–40%, 41–60%, and 61–100%. They used 20 impervious surface-canopy density combinations as input factors in the discriminant analysis of WNV bird infections. They interpolated the mean weekly precipitation for census block groups and added it as another variable (a total of 21 variables) to the analysis. Mean weekly precipitation and low to moderate percentage (0–20% and 21–40%) of impervious surface with 21–40% canopy density in the epidemiologic weeks of 26-37 in year 2002 were found to be positively correlated to bird infection. Liu *et al*. also used Mahalanobis distance statistic [128] to identify bird risk areas. Shorter distances indicate areas with higher risk of WNV in birds, and a larger distance associates with lower risk. They also found that distance to bird risk areas played a role in human infection. Housing density was found to be negatively associated with WNV dissemination, namely the higher the housing density, the less likely that birds have places to nest and the virus is less likely to be disseminated in such neighborhoods.

### 3.3. Spatial Analysis of Horses

One study, by Ward [62] examined the emergence of WNV in horses in counties located in northern Indiana in 2002. The purpose of his research was to use data from an outbreak of WNV encephalomyelitis in a population of horses to develop a temperature dependent spatial model. Locations for the confirmed cases were geocoded to particular locations and then a buffer was created to identify ten meteorological recording stations. The study period contained 106 days and for each day, the temperature was interpolated by an ordinary kriging function using Spatial Analyst in ArcGIS. They investigated the spatial progression of WNV. Their results revealed that the risk of WNV encephalomyelitis in this area was expected to increase rapidly during mid-August but then diminish during mid-September. Ward [62] demonstrated how GIS can be used to track the spread of an outbreak and predict future risk based on temperature and location. Ward *et al*. also assessed temporal relationship between equine and human cases in Texas in 2002 [129]. They found that equine cases in urban areas were reported significantly earlier than corresponding human cases. They suggested monitoring equine populations that are susceptible to WNV disease within close proximity to urban human populations.

Leblond *et al*. presented a spatio-temporal study of a recent outbreak in horses in Camargue, France [130]. The pixels of a SPOT-4 satellite image were classified into the following 14 landscape categories by a supervised classification method: open water, rice fields, reeds, cereals, woods, orchards, wet meadows, salt ponds, sparse and small elevation brackish (low and salted “sansouire”) swamps, dense and higher vegetation in less brackish swamps, dry lawns, dry bushes, bare ground. Using SatScan software [131], a Bernoulli model was applied for cluster identification. With this model, circles are centered in each location where information is available and the ratio of cases (confirmed WNV cases, horses with neurological syndrome) were compared to controls (horses with no neurological signs) for the area inside the circle with the ratio outside the circle. The environmental data for each landscape category belonging to each cluster were used for case-control analyses. Rice fields and dry bushes, wet “sansouire” and open water were the landscape categories most associated with the presence of WNV cases.

Lian *et al*. [132] used GIS and space-time cluster analysis software SaTScan to identify high-risk areas and shifts in disease clustering over a large geographic area in Texas. They focused on equine cases, which they argue are among the most susceptible to clinical WNV disease. Using data from 1,421 equine WNV case reports from six contiguous Health Service Regions, the researchers were able to identify potential high-risk areas for infection in these regions. ArcInfo was used to geocode cases to specific locations and then the regional database for these cases was exported and joined to the case point features. Spatial and Space-Time Scan Statistics were then used to detect unique non-random space-time clusters. They found that two distinct outbreak “waves” of an equine WNV epidemic could be identified, along with previously unrecognized areas of high risk. Lian *et al*. [132] also concluded that the 2002 epidemic did not spread uniformly, and local hot spots could also be found regionally within the larger epidemic. They argue that GIS and SaTScan can be effective tools for prevention and control of future epidemics by “indicating where and when limited resources can be used most effectively.”

### 3.4. Spatial Modeling of Mosquito Pools

Four studies addressed high-risk areas for WNV exposure to humans by focusing primarily on the spatial analysis of WNV-positive mosquito pools. Due to the significant health risk that WNV poses to the state of Mississippi, Cooke *et al*. [46] argued that GIS models can be used to create risk maps for the state. They analyzed both avian and environmental (land use, land cover, forest distribution, soils, and elevation) data to model the suitability for mosquitoes with WNV. Based on zip code boundaries, spatial depictions of human and bird occurrences were used for spatial analysis. The researchers found that WNV risk is correlated to high road density, low stream density, and gentle slopes. The results of their study found that WNV can be found in both urban areas as well as rural areas, which had generally been thought of as an urban problem.

Ozdenerol *et al*. [53] used GIS and spatial statistics to identify areas in Shelby County, TN (USA) that were ecologically most suitable for sustaining WNV. The researchers used GIS (ESRI products) and remote sensing (Erdas Products) to map locations where WNV-infected mosquitoes had been identified over different time periods and then used environmental variables, such as elevation, slope, land use (based on Landsat satellite image classifications), vegetation density, temperature and precipitation to help characterize areas containing WNV. Using raster-based data and Mahalanobis Distance statistics, they were able to create probability risk maps for areas ecologically most suitable for WNV-infected mosquitoes. The Mahalanobis distances allowed the researchers to quantitatively describe the landscape based on how similar it is to the “ideal” environmental conditions for infected mosquitoes. Based on probable ecological locations, they also compared social factors that may influence the transmission of WNV and found that characteristics such as high percentage of black population, low income, high rental occupation, old structures, and vacant housing are associated with the focal area of WNV infection cases. Ozdenerol *et al*. [53] argue that the techniques provided in this analysis can help better shape mosquito control strategies and can help with prevention efforts.

A study by Winters *et al*. [63] examined WNV in Colorado from 2003 and 2007 to determine how estimates of vector-borne disease occurrence are influenced by spatial scale of data aggregation. Data was aggregated to the census tract, zip code, and county units and statistical operations, such as hot-spot analysis and Spearman’s rank correlation were conducted. They determined that sub-county scale presentation can provide useful information for stakeholders.

A recent Australian study [133] predicted spatio-temporal spread of WNV in Australia over a 6 year period following the initial incursion at Sydney airport through the entry of an infected mosquito in an aircraft. U.S. incidence and fatality rates were used to estimate the potential number of human and equine cases in Australia. Six zones determined to be spread zones were overlaid on maps of human and equine populations. The total human population at-risk per zone was then used to estimate numbers of human cases of disease and deaths each year following a WNV incursion. The population at risk within each zone was discounted during each subsequent year by the number of cases that had occurred in the preceding year. 

### 3.5. Real-Time GIS Studies for WNV Surveillance

Four studies addressed real time GIS surveillance systems developed in Canada. A Canadian study by Gosselin *et al*. [118] developed a real-time GIS for public health surveillance of WNV in response to the first outbreak detected in the province of Quebec, Canada in 2002. This study did not explicitly describe the analytical methods used for monitoring and identification of areas for potential transmission to humans but rather how Quebec developed a public health plan that would help monitor human cases and look for ways of intervention. The Integrated System for Public Health Monitoring of West Nile Virus (ISPHM-WNV) includes information on Corvidae (American Crows, Blue Jays, and Common Ravens), mosquitoes, human cases, horses, climate, and preventive larvicide interventions and has graphical display (statistics), cartographic display (thematic maps), and management tools (administrative controls) components. The system uses a georeferencing tool to display and localize the monitoring data, and the cartographic interface allows for visualization and analysis. The population is invited, by means of media campaigns, to report the presence of dead Corvidae to a reporting center (WNV-Info line). Precise locations of corvid reportings were necessitated for collection and subsequent testing. Infected bird clusters were defined as potential risk zones. Mosquito surveillance took place in risk prone areas and at fixed monitoring stations. JMap software integrated cartographic data with cartographic navigation tools and spatial analysis functions and provides dynamic links of locations to multimedia documents. The authors conclude by explaining that this system has become an essential tool for field workers in all regions of the province, as well as for central authorities. It speeds up the delivery of relevant information to all actors and simplifies the task of data analysis.

In the United States, the CDC’s ArboNET, nationwide electronic WNV surveillance system monitors the geographic and temporal spread of WNV and reports data in several categories such as wild birds, sentinel chicken flocks, human cases, veterinary cases, and mosquito surveillance. Through this system, states share information that helps public health officials, elected officials and the public to develop national and regional public health strategies for WNV surveillance, prevention, and control [37].

In their paper, Jiangping *et al*. [134] has presented a real-time GIS-driven Surveillance pilot system for national WNV in Canada. This real-time system allowed users to track and monitor national West Nile Virus dead bird surveillance data for WNV risk protection, prevention, and control. After a 2005 outbreak of 224 clinical cases of human infection, with 12 deaths and WNV-positive dead bird sightings reported at five Canadian states (Ontario, Quebec, Manitoba, Saskatchewan, and Alberta), a nationwide electronic surveillance was developed by the Centre for Food-borne, Environmental and Zoonotic Infectious Disease under the Public Health Agency of Canada (PHAC)*.* This system produces weekly WNV monitoring reports and maps, summarizing WNV activity during the WNV season, which is May through October [135].

Bouden *et al*. [70] reported the WNV-Multi-Agent Geo-Simulation (WNV-MAGS) Project in his paper. The MAGS System was developed by Dr. Moulin’s laboratory in Laval University in Quebec City, Quebec, Canada. This system helps with short term decision-making related to use of larvicides based on climatic scenarios and the characteristics of the environment.

### 3.6. Habitat-Based Studies

Ten articles examined the quantification of vector-host interactions and defined the suitability of various landscape features and meteorological variables for mosquito transmission. Two of these articles integrated spatial analysis with computational neural network approaches.

Four of these studies were led by Jacob [39,40,60,61]. The first study examined habitat characteristics of two mosquito vector species *Culex pipiens* and *Culex restuans* in residential areas of Urbana-Champaign, Illinois [39]. The result of their LULC classification of Landsat Data revealed a 12.1% change in LULC types between 1991 and 2003 in Urbana-Champaign, Illinois. 20,166 egg-rafts were sampled in maintained urban (76.7%) and in maintained non-urban (14.8%) areas. 56.4% of the total sample was harbored by the well-drained stratum. An analysis of remotely sensed imagery Quick bird visible and near infrared data revealed that the egg-rafts were distributed mostly in residential high canopy coverage areas (83.3%). A second study by Jacob *et al*. applied geospatial modeling to predict adult abundance of *Culex* species in a mosquito abatement district of northern Cook County, Illinois [60]. WNV infection rates were sampled weekly during the season May to October between 2002 and 2005. Their multivariate analyses results revealed that forest along with middle range built environment predicted heightened infected vector activity. The two mosquito vector species *Culex pipiens* and *Culex restuans* abundance was positively associated with temperature and negatively associated with precipitation. A third study by Jacob utilized multispectral satellite (Quickbird) data and DEM-based GIS analysis to determine drainage basin physiographic predictors associated with *Culex erraticus*, a mosquito vector of eastern equine encephalitis virus (EEEV) and Northern cardinals, an avian host associated with WNV in Tuskegee, Alabama. They found significant inverse linear relationship between the *Culex* adult count data and elevation [61]. Jacob and associates further used high resolution WorldView-2 (WV-2) satellite imagery for habitat analysis of mosquito vector specie *Cx. Quinquefasciatus* in Trinidad [40]. Through DEM-based GIS analysis, they derived terrain-related parameters. The result of their predictive geospatial models using field and remote-sampled explanatory variables of *Cx. Quinquefasciatus* habitats revealed that Terrain elevation was significantly associated with the *Cx. Quinquefasciatus* habitats sampling sites. The sampled habitat distributions based on egg-raft counts displayed positive autocorrelation.

One U.S.-wide study by Soverow [136] investigated meteorological conditions associated with reported human WNV cases in the United States. Soverow *et al*. studied the effects of ambient temperature, humidity, and precipitation on the incidence of WNV infection among 16,298 cases reported to the CDC between 2001 and 2005 in 17 U.S. states. They used case-crossover study design to evaluate the association between meteorological variables and WNV cases. The primary outcome measures were the incidence rate ratio of disease occurrence associated with mean weekly maximum temperature, cumulative weekly temperature, mean weekly dew point temperature, cumulative weekly precipitation, and the presence of less than or equal to 1 day of heavy rainfall greater than or equal to 50 mm during the month prior to symptom onset. Their results revealed that warmer temperatures, elevated humidity, and heavy precipitation each increased the rate of human WNV infection in the United States independent of season. The Intergovernmental Panel on Climate Change as cited in Soverow projects that climatic and weather conditions in North America in the coming decades are likely to include warmer temperatures, shorter winters, increased proportion of precipitation falling as rain rather than snow, and increased frequency of heavy rainfalls and other extreme weather events. Based on Soverow’s results, such changes may increase the burden of this disease in coming decades.

Another Canadian Study by Tachiiri *et al*. [48] applied raster-based approach to map *Cx. tarsalis* abundance and identified WNV risk prone areas in British Columbia. Their spatially-explicit mosquito abundance model revealed the highest potential abundance in five regions in British Columbia (Okanagan Valley, the Thompson Region, Greater Vancouver, the Fraser Valley, and southeastern Vancouver Island).

Epp *et al*. [2] conducted a study in rural Canada to predict areas of low, medium, and high risk of West Nile Virus (WNV) in humans in both 2003 and 2007 in the province of Saskatchewan. Land surface temperature, precipitation, vegetation cover, and LULC (classified from satellite imagery) were incorporated into a discriminant analysis model with the production of risk maps as an end product. Information from modeling of horse and human surveillance data conducted in Saskatchewan in 2003–2005 were used to create a historical training dataset. Comparison of the 2007 yearly and historically trained models was done with the kappa statistic. Risk maps from the historically trained 2007 model revealed a southwest to northeast decreasing trend of risk.

A recent Moroccan study [137] compared the differences between eco-climatic conditions (temperature, rainfall, and vegetation index) recorded during epidemic years (2003 and 2010) and those recorded during years with no apparent WNV cases. WNV was reported in equines living in the central and north-western part of the country. They used remotely sensed datasets such as MODIS Land Surface Temperature and Emissivity (MOD11A2), MODIS vegetation Indices (MOD13Q1) and precipitations (TRMM-Tropical Rainfall Measuring Mission project). A circular area of 10 km radius was arbitrarily drawn around each case location, and the mean value of all pixels falling within each polygon for each NDVI image was calculated. NDVI values (an indicator found to be reliable to assess precipitation in Morocco) were significantly higher in the epidemic years, whereas temperature did not play a crucial role in Morocco.

Ghosh and Guha [138] used genetic algorithm (GA) and computational neural network approaches (non-linear machine learning techniques) to develop a WNV neural network model. They used WNV-infected bird data for the year 2006 to build the model. The built environment, proximity to features and climate variables were aggregated at the zip code level. They used GA to identify the best subset of predictor variables. By computing neural networks (CNN), the predictor variables are ranked in order of their importance to predict the number of WNV-infected dead birds. The CNN model correctly predicted the number of infected birds for 74 zip codes. The spatial distribution of the observed and predicted numbers of infected dead birds validated the same pattern.

Ghosh and Guha [139] conducted another study expanding the application of CNN models as both predictive and explanatory tools. They proved that CNN models were better suited to capture the non-linear relationships with greater accuracy than linear regression models. They analyzed how proximity or spatial distribution of a particular risk factor influences the risk of WNV. They found that with closer proximity to bogs there is a higher risk of WNV infection. Their finding was critical for targeting chemical control vector abatement programs, such as larviciding and adulticiding.

### 3.7. Remote Sensing Studies for Early Warning Systems and Vector Control

Recently RS-driven studies have been used for WNV early warning systems. By identifying high-risk areas in advance, public health officials can improve effectiveness of disease prevention measures and minimize pesticide usage by integrated early warning systems using satellite data. For example, Liu *et al*. conducted three studies employing ASTER imagery assessing environmental conditions and WNV dissemination at the local scale [127,140,141]. These studies fused high spatial-resolution ASTER and high temporal-resolution MODIS images to derive urban environmental variables (normalized difference vegetation index, normalized difference water index, and land surface temperature (LST)) with mosquito surveillance records during desired epidemiologic weeks. By using the Spatial and Temporal Adaptive Reflectance Fusion Model (STARFM) image fusion model, they simulated ASTER and MODIS surface reflectance images. The fused image datasets allowed for estimating environmental parameters during preferred time periods such as the desired epidemiologic weeks. They found that the elevation and urban built-up conditions were negatively associated with the WNV propagation, while LST was positively correlated with viral transmission.

Chuang *et al*. [142] conducted a study comparing environmental parameters derived from NASA advanced microwave scanning radiometer on EOS (AMSR-E) and *in situ* weather station data to predict abundance of mosquito species *Aedes vaxans* and *Culex tarsalis* in Sioux Falls, South Dakota. Despite its coarser resolution (25 km), the AMSR-E-derived models had better forecasting accuracy than weather station data. They found that air temperature and vegetation opacity were the best tested predictors of *Cx. tarsalis* abundance, whereas air temperature and surface water fraction were the best predictors of *Aedes vaxans*. This research concluded that passive microwave radiometry proved useful to estimate global surface air temperature, and was largely insensitive to atmospheric contaminations, under cloudy, non-precipitating, non-frozen conditions.

Cleckner *et al*. [143] calculated a Tasseled-Cap transformation from a July 2002, Landsat 7 Enhanced Thematic Mapper (ETM+) image to capture spatial and spectral habitat variation for modeling mosquito abundance in Coastal Virginia. Using the Tasseled-Cap transformation, they were able to separate brightness (correlated with texture and moisture content of soils), greenness (vegetation density) and wetness bands (moisture in soils, vegetation and other surface cover) to quantify environmental variables for a potential habitat suitability index.

Thompson *et al*. [144] utilized an Airborne Visible Infrared Imaging Spectrometer instrument (AVIRIS) to compare spectral attributes of pool conditions with colonization of the *Culex* Species. Mosquito colonization of unmaintained pools coincides with accumulation of spectrally distinctive algae. This technique is compared to a technique developed by Kim *et al*. [145] who located pools using GeoEye satellite images, detecting pools from a Normalized Difference Water Index (NDWI). Green pool bottom could be mistaken for algae in images using visible light images only. Spectroscopic data overcomes this by revealing the unique spectral signatures of algal pigmentation and can directly be used to estimate the probability of colonization, an effective tool for vector control.

### 3.8. Spatial Analysis of Genetic Variation

Studies of phylogeographic analysis with spatial statistical modeling have yielded valuable insights into broad patterns of WNV evolution and emergence at coarser scales [111,146,147]. Bertoletti *et al*. improved his earlier research [111] by applying population genetic analyses at a finer scale to correspond with localized environmental conditions. His results clearly demonstrated that WNV varies genetically over geographic and temporal scales that are finer than had previously been appreciated [148]. They compared nucleotide diversity for viruses from birds and mosquitoes from both natural and residential sites in suburban Chicago. They found viral transmission was distance-related and varied seasonally in response to fine-scale microclimatic and landscape characteristics related to urbanization.

### 3.9. Spatial Uncertainty Analysis

Wey *et al*. [149] investigated spatial uncertainty and selection bias in the ability to geocode WNV cases in South Dakota. They compared spatial patterns of geocoded and non-geocoded cases and examined the influences of population density and Indian reservations on geocoding success at a zip code tabulation area (ZCTA) level. They used the spatial scan statistic for cluster analyses at the ZCTA level. They separated their dataset into two groups: epidemic years (an increase in the prevalence of a disease over baseline rates) and endemic years. They reported encountering a lower ability to geocode individuals on reservations. They also reported a significant difference in the proportion of cases that could be geocoded, with a larger percent of cases not being geocoded during the epidemic years.

## 4. Conclusions

Fourty-seven GIS-based studies examining spatial modeling of WNV infection and risk factors have been published since the New York City outbreak in 1999, and they indicate a growing geographic spread of the virus and the importance of climatic factors in addition to biological and ecological determinants on WNV occurrence. Furthermore, significant associations have been found between WNV infection and some socio-economic and microclimatic indicators, though these seem to be location-dependent. Recent finer scale microclimatic studies [142,143,144,145,146] applied at various temporal scales re-emphasize the extent of the role and precision of risk factors and the likely influence they are having on the rapidly growing burden of WNV in the world. Surveillance methods that accommodate spatial analysis at various temporal scales have potential to provide understanding of location-dependent risk factors. Fusing high temporal-resolution and high spatial-resolution remote sensing imagery can offer higher predictive accuracies for estimating location-dependent microclimatic conditions associated with WNV occurrence. Making use of such newly available remote sensing products improves quality of surveillance data and can lead to tangible improvements in the prevention and control of WNV.

There is a growing trend for WNV research to move from descriptive towards predictive studies and more exploratory investigations. Public health officials are increasingly challenged to assess the prevalence and to determine the most common risk factors, as well as to track their trends over time. Very few studies have made the leap from spatial prediction to risk-based surveillance [69,118,136]. Deviation from use of linear models to more powerful nonparametric models such as computational neural networks that capture non-linear relationships with greater accuracy have more applicability in prevention and control, especially for chemical control vector abatement programs [138,139]. Real-time studies provide reliable predictions but depend on high-quality input data. Many datasets utilized in such analyses are not collected for such spatial analytical purposes and have limitations with respect to spatial coverage, temporal appropriateness, and precision, accuracy of georeferencing and geocoding success. There is only one study published regarding selection bias in the ability to geocode WNV cases [149]. Future work lies in strengthening data collection techniques and eliminating some of the inherent spatial uncertainties in this field of research.

In our opinion, studies of phylogeographic analyses need to be further integrated with spatial statistical modeling and accommodate subsequent analysis at various scales. Their applicability in real-world decision-making needs to be investigated, especially in localized environmental conditions.

Overall, spatial epidemiology studies in this review maintained evidence that WNV has become epidemic in North America and those annual outbreaks are likely for the foreseeable future. It has reached Central and South America, though analysis of WNV distribution and public health impacts remain poorly understood in these regions. WNV is known to have reached western China (Xinjiang Province), but there is no evidence of WNV in the rest of China: however, surveillance reports do not exist from most of China, so there is little knowledge about the possible distribution of WNV in China. Reemergence of the virus in India is a threat to highly populated countries of Southeast Asia. Recent confirmed cases in Japan and South Korea prompted these countries to start nationwide WNV surveillance efforts and international collaboration of intense research efforts to determine the particular strains involved.

West Nile has been appearing more frequently in the Mediterranean and Eastern European nations in recent years. Genome sequence analysis of WNV strains isolated in Italy in 2008 and 2009 showed they were closely related suggesting the virus had overwintered and established an endemic cycle. In 2012, early detection of human cases of WNV disease has been reported also in Sardinia [26], in Greece, Israel, and the occupied Palestinian territory [150], and this trend might predict increased viral activity in the Mediterranean area.

The WNV activity in southern Europe suggests that the avian hosts contribute to the movement of the virus. The virus is translocated by the viremic migratory birds to the temperate and tropical regions and more likely persist in the southern wintering sites in the Western Hemisphere [57]. Future GIS-based ecological studies are needed to examine the frequency of cycling of active virus in the avian hosts and detect the favorable habitat and climatic conditions for the migrating vectors. Future studies should also consider whether the local vectors are capable of transmitting the virus.

The prospective techniques are mainly early warning approaches based on information on a single component of the WNV transmission cycle, *i.e*., either infected birds or human cases. Some studies found that positive bird information does not appear to offer much predictive value in spatial models [126], while others have reported successful prediction of human risk with this approach [50]. The dynamic distribution of bird populations, sampling bias (due to voluntary public surveillance) and other factors could all complicate any meaningful associations between WNV infection in humans and birds. More vegetation means increased habitats for both WNV vector and bird reservoir hosts, with urban green areas having the necessary tree cover to support bird populations and contact between migratory and residential bird species, which has been found to be important in WNV amplification [57]. Lower overall land-cover diversity may indicate greater concentration of bird species that are efficient hosts for the virus, and thus the potential for higher incidence of WNV in humans [57]. Future studies on land-cover diversity and avian cases need to be conducted.

Large and dense groups of migratory birds gather in wetlands and potentially reach every part of the southeastern United States, Mexico, Central America, the Caribbean Island and South America during their migration south to wintering sites and nearly every part of North America during their migration north to breeding sites [57]. The suitable wetland sites along the coastal plain of Georgia, northern Florida, and Alabama are likely to receive the largest number of potentially infected hosts, and therefore may be the most likely places for future outbreaks, if the necessary ornithophilic mosquitoes are sufficiently active and abundant [57]. Future spatial-temporal analyses on intense monitoring of fall and winter avian concentrations for abnormal die-offs are highly suggested in the coastal areas.

More measures on improving the representativeness of bird surveillance data should be taken. Areas with low human population and low bird densities may choose to focus surveillance efforts on mosquito and human cases. Media communications should encourage the public to increase their reporting of dead bird sightings.

Improved surveillance efforts of avian and mosquito species across metropolitan regions as well as prevention controls in reducing breeding grounds will have a significant impact on reducing the risk of WNV in humans. The investigation of reservoir hosts and vectors is the key to determine WNV risk prone areas and will be the focus of future work. The assessment risk of WNV to humans cannot be made outside of the context of the urban environment in which it is present. The effect of mosquito control is apparent. Future research in the response of residents to the risk of WNV and a qualitative assessment of the political and social factors related to mosquito control should also be carried out. Effective public outreach and health education are key factors to eliminate WNV risk to humans. This review excluded these studies since focus was on geographic distribution and spatial modeling.

Future research should be concerned with the noticeable change in pattern and magnitude of human WNV outbreaks between years. It is possible that various mosquito control efforts (and lack thereof) on the part of political entities may have an effect on the patterns of human WNV illness, and these impacts have not been studied well. Seasonal climate and temperature patterns and soil moisture characteristics play a part in *Culex* abundance and WNV transmission, and should also be explored more thoroughly. Storm water systems also may be particularly suitable for vector production. Catch basins often provide the stagnant water and the cool moist environment needed by *Culex* to survive in hot dry weather and deposit their eggs, and an exploration of catch-basin characteristics and their locations would be a valuable contribution toward increased understanding of WNV transmission. Delineation of WNV transmission cycles at local scales can be used to develop hypotheses related to the disease spread and thus advance our understanding of the complexity of avian-mosquito-human environmental systems on a micro-scale. The modes of transmission such as blood transfusion need to be revisited through public health studies.

Continued development in satellite remote sensing offers frequent worldwide coverage in high spatial and spectral resolutions appropriate for assessing vegetation and moisture conditions at local and intermediate scales, including the availability of historical images comparable with historical WNV data. Remote sensing can be used more often and more effectively in assessing WNV-carrying mosquito habitats over time and space.

More empirical knowledge on the spatiotemporal patterns of mosquitoes is needed. For example, *Cx. Quinquefasciatus* larval habitats and their underlying contribution to adult population are needed to devise an effective WNV surveillance program in regions of the Southern United States and Caribbean [40]. Habitat-based interventions should emphasize the link between foraging behaviors of egg-laying mosquitoes and the availability of breeding sites in evaluation of environmental management programs. 

More research is needed to assess the effects of expected climate shifts on WNV transmission and studies need to be designed on longer time scales. Shorter time scale studies also have strengths that include the ability to restrict variables to one year or season, which can improve the power to study geographic variation, the age immunity *etc*. status of human cases, socioeconomic characteristics, linking vector to reservoir host populations, and more.

GIS-based ecological studies have demonstrated how GIS can be used to better understand WNV to track outbreaks and to create prevention and control techniques. Additionally, spatial statistics are also very useful when determining relationships between WNV cases and other variables, such as ecological or social factors. We need to improve our understanding of the extent spatial-temporal methodologies can be applied to other WNV-prone areas of the world, given that the characteristics of transmission cycle and surveillance programs vary significantly from one region to another.
